# The Gut-Brain Axis in Autoimmune Diseases: Emerging Insights and Therapeutic Implications

**DOI:** 10.7759/cureus.48655

**Published:** 2023-11-11

**Authors:** Muhammad Muneeb Khawar, Sami Ijaz, Priya Goyal, Dhanuddara Kandambige, Mouhammad Sharifa, Abdalkareem Nael Jameel Maslamani, Salem Al Kutabi, Inam Saleh, Mohamed Mustafa Albshir, Mohammed Khaleel I KH Almadhoun, Sana Nazir Soomro, Neelam Kumari

**Affiliations:** 1 Internal Medicine, Mayo Hospital, Lahore, PAK; 2 Internal Medicine, North China University of Science & Technology, Tangshan, CHN; 3 Internal Medicine, Dayanand Medical College & Hospital, Ludhiana, IND; 4 Internal Medicine, Kursk State Medical University, Kursk, RUS; 5 Medicine, Faculty of Medicine, University of Aleppo, Aleppo, SYR; 6 General Practice, Cairo University, Cairo, EGY; 7 Medicine, Cairo University, Cairo, EGY; 8 Pediatrics, University of Kentucky College of Medicine, Lexington, USA; 9 Internal Medicine, Bahri Teaching Hospital, Khartoum, SDN; 10 Medicine and Surgery, Mutah University, Karak, JOR; 11 Internal Medicine, Jinnah Medical & Dental College, Karachi, PAK

**Keywords:** general internal medicine, gastro-intestinal surgery, gut, gut-brain axis, gut-brain connection

## Abstract

The gut-brain axis (GBA) is a two-way communication system that is influenced by signals from the nervous system, hormones, metabolism, the immune system, and microbes. The GBA may play a key role in gastrointestinal and neurological illnesses. Signaling events from the gut can regulate brain function. As a result, mounting data point to a connection between autoimmune disorders (AIDs), both neuroinflammatory and neurodegenerative diseases, and the GBA. Clinical, epidemiological, and experimental studies have shown that a variety of neurological illnesses are linked to alterations in the intestinal environment, which are suggestive of disease-mediated inter-organ communication between the gut and the brain. This review's objective is to draw attention to the clinical and biological relationship between the gut and the brain, as well as the clinical importance of this relationship for AIDs, neurodegeneration, and neuroinflammation. We also discuss the dysbiosis in the gut microbiota that has been linked to various AIDs, and we make some assumptions about how dietary changes such as prebiotics and probiotics may be able to prevent or treat AIDs by restoring the composition of the gut microbiota and regulating metabolites.

## Introduction and background

Over three decades have passed since the introduction of the gut-brain axis (GBA) theory [1]. The gut-brain axis refers to the two-way communication between the brain and gut bacteria [2-5]. Involving nutrients, hormones, and afferent/efferent regulation autonomous neuronal networks, the GBA symbolizes intricate interactions between the gut and brain. Numerous physiological processes, such as satiety, control of glucose and fat metabolism, hormone secretion and sensitivity (particularly insulin sensitivity), and bone metabolism, are regulated by the GBA. This axis also involves the immune system, autonomic and enteric nervous systems, hypothalamic-pituitary-adrenal axis (HPA), microbiota, and its metabolites [3,6,7]. Immune-stimulatory signals from the intestinal microbiome may trigger both innate and adaptive immune responses, and it has been demonstrated that the adaptive immune system regulates the diversity and composition of the intestinal microbiome. Therefore, the immune system is crucial for the dynamic balance between the gut and all major organs, including the brain. It is theorized that many diseases are linked to alterations away from a "healthy" gut microbiome because of the physiological interactions between humans and our microbial ecosystems. These include cancer, metabolic disorders, inflammatory and autoimmune diseases, and neurological ailments. Autoimmune diseases are mostly influenced by genetic predisposition, environmental factors (including infections), and gut dysbiosis. Autoimmunity develops gradually, manifests as circulating autoantibodies in the peripheral blood, and can be identified several years before clinical illness. Altered gut microbiota can cause autoimmune diseases through molecular mimicry, disordered metabolomes, and the migration of pathobionts and their metabolites, such as lipopolysaccharides (LPS). This is a comprehensive review of the current knowledge on the intricate relationship between GBA and autoimmune disorders (AIDs). In particular, we focused on the underlying mechanisms and pathways that link the gut and brain in AIDs. This involves exploring how factors such as gut microbiota, immune system dysregulation, and neuroendocrine signaling contribute to autoimmune disease development. Moreover, microbiota-based therapeutic strategies for GBA-associated brain and AIDs are also discussed.

## Review

Gut microbiome and autoimmunity

The term "microbiome" encompasses all microorganisms and their associated genetic material that inhabit the human body. In contrast, "microbiota" pertains to the collection of microorganisms residing in particular regions, such as the skin or gastrointestinal system. In the last 10 years, it has become abundantly obvious that the gut microbiota plays a crucial role in controlling the GBA. The gut is home to trillions of different microorganisms, mostly bacteria but also yeasts, viruses, helminth parasites, and protozoa [8-13]. Bacteroidetes and Firmicutes are the two most prevalent phylotypes in the bacterial gut microbiome, with Proteobacteria, Actinobacteria, Fusobacteria, and *Verrucomicrobia phyla* present in comparatively low quantities [8,13,14]. Even among healthy individuals, the microbiota composition varies greatly [3,15]. In a healthy host, the microbiota affects a wide range of physiological functions, including the immune system, development of various host organs, food digestion and absorption, and defense against pathogens [3,16-19].

The physiology and pathology of the host are fundamentally influenced by the human microbiota. It has been demonstrated that the gut microbiota and its metabolites have a systemic impact on immunological processes and immune homeostasis [20]. It has been demonstrated that short-chain fatty acids (SCFAs) generated from microorganisms and biotransformed bile acids can affect the immune system by serving as ligands for particular cell signaling receptors or by altering the immune system's epigenome. Bacterial translocation is a significant cause of chronic systemic inflammation and, with leaky gut (intestinal permeability), may operate as a constant source of inflammation that might lead to autoimmune responses [21]. An effective approach to illustrating the microbiota's role in shaping both innate and adaptive immunity involves employing germ-free (GF) models. These models entail raising animals in completely sterile environments, ensuring they have never encountered any microorganisms [20,22]. As an alternative, altering the microbiota through antibiotic therapy or microbiota genetics also offers important proof of the microbiota's function in immunological homeostasis [20,23-28].

Dysbiosis is characterized as a reduction in the diversity of gut bacteria as a result of a change in the ratio of commensal to possibly pathogenic microorganisms [29-33]. It is a condition linked to illnesses that affect other distal organs in addition to gastrointestinal conditions. Recently, it was discovered that the microbiota might alter the physiology and cause inflammation of the central nervous system (CNS) through its GBA, which has several connections, including the vagus nerve, the immune system, and bacterial metabolites and products.

The intestinal wall serves as a barrier to the external environment, preventing the entry of pathogens and harmful substances [34,35]. It has been established that intestinal barrier function is significantly regulated by the gut microbiota [36]. Dysbiosis can disrupt this protective barrier, causing heightened permeability of the intestinal lining to gut bacteria or other substances in the gut lumen. This can potentially trigger molecular mimicry, a widely recognized mechanism in the development of autoimmune disorders. The autoimmune disorders caused by dysbiosis (altered gut microbiota) are outlined below.

Gut Microbiota and GI-Associated Autoimmune Disease

Inflammatory bowel disease (IBD): Inflammatory bowel disease is an autoimmune disorder that affects the GI tract and primarily presents in two major forms: Crohn's disease and ulcerative colitis [20]. There is strong evidence that bacteria play an important role in IBD pathophysiology. For instance, antibiotic therapy is frequently beneficial for IBD patients as well as animal models of the disease [37-39]. In addition, healthy adults with IBD have very different gut microbial phyla [32,37]. However, intestinal inflammation has been reported to be the primary contributor to dysbiosis, which favors the selection of microbiota species with a colitogenic phenotype. Another study utilizing mice with inflammasome pathway deficiencies emphasized the significance of inflammation in disease and dysbiosis. Several different nucleotide-binding oligomerization domain-like receptor proteins (NLRP) form cytoplasmic multiprotein complexes known as inflammasomes, which serve as sensors for stress events. The gut microbiota altered in NLRP-I mice due to a change in the inflammasome pathway causes a notable increase in *Prevotella *and TM7 species, thus making NLRP-I mice vulnerable to colitis [37,40].

Gut Microbiota and Extraintestinal Autoimmune Disorders

Rheumatoid arthritis (RA): Rheumatoid arthritis is a chronic autoimmune condition characterized by joint inflammation and damage. The etiopathogenic mechanisms involve the interaction of innate and acquired immune responses, antigen-presenting cells (APCs), the development of self-reactive T cells (Tregs), and the production of autoantibodies, such as rheumatoid factor and anti-citrullinated protein antibodies (ACPAs) [41]. Recent investigations have shown a connection between gut microbiota and autoimmune conditions using an autoimmune K/BxN arthritis model [26,37]. A transgene-encoded T cell receptor that responds to a self-peptide is expressed in K/BxN mice. When segmented filamentous bacteria (SFB) colonizes the gut, Th17 cells differentiate. These cells then leave the gut and enter the peripheral lymphoid tissue [37,42]. In turn, interleukin (IL)-17 acts directly on B cells to aid in germinal center B-cell development and the generation of autoantibodies in the spleen. Subsequently, the autoantibody travels to the joints of the target organ, eventually causing illness.

Type 1 diabetes (T1D): Type 1 diabetes is an autoimmune disorder characterized by the gradual destruction of autologous beta cells in the pancreas that produce insulin. Genetic, epigenetic, and environmental variables affect the likelihood of developing T1D. Birth delivery method [43-45], food during infancy [43, 46-49], and potential antibiotic use [50, 51] may influence the risk of developing T1D. These potential environmental risk factors are connected to the intestinal microbiota.

Gut microbiome makeup and the abundance of specific bacteria appear to be the main determinants of diabetes development. One of the key characteristics of the clinically silent phase before the onset of T1D is the presence of microbiota with few commensal butyrate producers, which has a deleterious impact on gut permeability [52-56]. The reduction in intestinal permeability results in the migration of microorganisms and microbial metabolites and may cause the activation of immune cells. Patients with T1D show a large decline in intestinal Tregs, suggesting that the gut microbiota may be involved in the disease [37,57]. Although several autoimmune models tend to show reduced disease severity in GF environments, T1D stands out as a notable exception. Another study found that non-obese diabetic (NOD) mice did not develop diabetes in a specific-pathogen-free (SPF) environment, suggesting that the gut microbiota may have a protective function [37,58].

Multiple sclerosis (MS): Multiple sclerosis is an autoimmune demyelinating disorder of the CNS. Gut microorganisms talk to the CNS through a two-way communication channel called the gut-brain axis. Cytokines and other immune cells are released when the gut microbiome is disturbed, which impacts the blood-brain barrier (BBB) and intestinal permeability. Recent studies utilizing animal models have shown that the gut microbiota may have a significant impact on the pathophysiology of experimental autoimmune encephalomyelitis (EAE)/MS. Demyelination, axonal loss, diminished oligodendrocyte numbers, gliosis, and astrocyte activation are all aspects of MS pathophysiology. The mechanisms through which dysbiosis is associated with MS are explained in detail below.

Mechanisms underlying gut-brain communication

The GBA is responsible for sending messages from the muscular and sensory components of the digestive system to the brain [1,59]. Gastrointestinal signals are thought to be transmitted to the brain via a variety of multichannel sensing and trafficking routes, which form the foundation of the gut-brain axis. These mechanisms are orchestrated through vagal and spinal afferent neurons, the neuroendocrine-HPA axis, and the immunological system, as depicted by a multitude of cytokines, neurotransmitters of microbial origin, and intestinal and cerebral barriers [60].

The gut microbiota communicates with the CNS through various mechanisms. These methods involve direct stimulation of the vagus nerve, leading to the release of acetylcholine or catecholamines [61,62] and interactions with enteroendocrine cells, resulting in the production of diverse neuropeptides as well as gut hormones, neurotransmitters, or microbe-associated molecular patterns (MAMPs). Furthermore, the gut microbiota produces microbial compounds capable of triggering immune cells to release cytokines, facilitating various forms of immune cell activation, even in peripheral areas. For instance, SCFAs can stimulate dendritic cells (DCs) [61,63,64] to generate retinoic acid (RA) [61,65], inhibit histone deacetylases (HDACs), and induce regulatory T cells [61,66,67]. This implies that dietary factors may play a role in the intricate interplay between the gut microbiota and immune system, which underpins neuroinflammatory diseases such as MS.

Additionally, Rutsch et al. described how the innate immunological inflammasome pathway may occasionally serve as a channel of communication between microorganisms and the central nervous system [3]. The deregulation of these physiological pathways might result in the overactivation of the inflammasome in the brain. Astrocytes [3,68], oligodendrocytes [3,69], endothelial cells [70], neurons [71], microglia, the brain's essential innate immune cells [72], and perivascular brain-resident macrophages [73] have all been implicated in inflammasome signaling in the CNS. Owing to the microbiota, inflammasomes are constantly stimulated in the intestine under physiological conditions [3, 74]. The intestinal inflammasome can be activated by various microbes, which can have an indirect impact on the brain. Inflammasome activation has primarily been associated with neuroinflammatory disorders of the CNS. For instance, it is crucial to the development of various neurological diseases such as MS, Alzheimer's disease (AD), postpartum depression (PPD), and neuropsychiatric symptoms (NPS) [3, 40, 74-78]. According to certain theories, the microbiota-gut-brain axis may also use inflammatory pathways to mediate the development of depressed behavior. Cognitive decline, autism spectrum disorder (ASD), and neurobehavioral symptoms of obesity are neurological diseases that coexist with intestinal inflammation and proinflammatory serological profiles that correlate with cognitive and behavioral deficits [79, 80].

The human diet contains a wide range of nutrients, including various kinds of carbohydrates and lipids. Changes in the ratio of certain dietary elements, such as the current common consumption of excessive fat, salt, and fiber, have been linked to autoimmune disorders, including MS [61]. Polyunsaturated fatty acids [61,81], vitamin D [61,82], and a Mediterranean diet [61,83] have all been linked to beneficial effects. Excessive salt consumption has been shown to increase pro-inflammatory T helper cell subsets [84-86], which in turn worsens disease severity in several animal models [61, 87]. Short-chain fatty acids, comprising three to five carbon atoms, promote the activation of Tregs, leading to the alleviation of the condition. Conversely, medium- and long-chain fatty acids consisting of more than 12 carbon atoms trigger the activation of pro-inflammatory T cells and exacerbate CNS autoimmunity [61, 88].

Autoimmune diseases associated with the GBA

In the last 15 years, the swift progress in microbiome science has brought about a shift in perspective. We have moved away from the traditional focus on the GBA and now embrace a systems biology approach to understand the intricate interactions within the gut-brain-microbiome system. This new perspective acknowledges bidirectional communication between the brain and the gut, encompassing aspects like the enteric neuroendocrine system (ENS) and the gut microbiome [89]. Multiple sclerosis is an inflammatory disorder characterized by the demyelination of nerve cells in the CNS [90, 91]. It is an autoimmune disorder that affects the CNS. Experimental autoimmune encephalomyelitis (EAE) is an animal model for human MS. Since EAE is not a naturally occurring model and requires the use of the bacterial adjuvant CFA for disease induction, the underlying disease mechanism of EAE may differ greatly from that of human MS [20]. 

It has been shown that MS/EAE patients exhibit dysbiosis [92, 93] and increased intestinal permeability, possibly as a result of changes in the architecture of the intestinal epithelial barrier. These modifications allow bacteria or their byproducts to migrate from the gut lumen into the lamina propria, causing inflammation [94-99]. This causes the migration of encephalitogenic T lymphocytes [92, 100] to the CNS and causes inflammation. This neuroinflammation may be the cause of MS/EAE. Changes in the vascular permeability of the BBB may lead to neuroinflammation. The rapid activation of microglia caused by the seepage of plasma elements such as fibrinogen, followed by the influx of inflammatory cells and signaling to the gut, may be the cause of the disease [92, 101-103]. This, in turn, causes intestinal barrier permeability [104] and feeds the brain-gut inflammation cycle.

In this context, it's worth noting that research has shown that intestinal CD4+ T cells may develop CNS autoimmunity and that T-cell activation occurs in the gut [92,96,105]. A recent study also identified a connection between the presence of the TGF inhibitor Smad7 on intestinal CD4+ T cells and autoimmunity in EAE [106]. The differentiation of naive CD4+ T cells is regulated by TGF- and Smad7 and Smad7 has been specifically linked to Th1-cell responses in both MS and EAE [107]. Parodi and Rosbo reported a shift in TGF-dependent Th-cell differentiation patterns in the lamina propria, marked by an increased presence of inflammatory T cells and decreased FoxP3 expression [92]. This pattern resembles the impaired TGF-1/Smad signaling observed in IBD [108]. In animal models of MS, there is a growing acknowledgment of the substantial involvement of gut immune cells as important contributors to CNS autoimmunity [92]. As a result, the gut is an anatomical location that may link immune control, the microbiota, and autoimmunity.

Therapeutic implications 

Immune-modulating medications and immunosuppressants, such as corticosteroids, are two types of current treatments for patients with MS [109]. In general, the former seeks to slow the deterioration of the neural system, whereas the latter frequently works to alleviate symptoms to help preserve quality of life.

Oral Antibiotics

Clopitts et al. demonstrated that oral broad-spectrum antibiotics can significantly reduce the risk of EAE by modifying the gut microbiota [110, 111]. The positive outcomes of oral antibiotic therapy have been described in numerous other studies. Yokote et al.'s [112] innovative combination of antibiotics successfully decreased EAE illness. They discovered that a subgroup of invariant natural killer (NK) T cells was necessary for the effectiveness of oral antibiotic therapy.

A rat model of EAE has also been treated with minocycline, an oral tetracycline antibiotic frequently used to treat acne, to lessen the severity of the condition both preventatively and therapeutically [109, 113]. Minocycline has since been used to treat MS patients in three clinical trials, either alone or in conjunction with other treatments. Minocycline has been shown to lessen CNS impairment when administered to individuals along with glatiramer acetate [109, 114]. In the future, alternative oral antibiotics or combinations of antibiotics could be explored to promote anti-inflammatory interactions within the gut-brain axis, regardless of whether minocycline ultimately demonstrates its effectiveness in the context of MS.

Probiotic Usage

Any live bacterium that offers a considerable health advantage to the host is a probiotic. Both commensal microorganisms, which are naturally found in the gut, and exogenous, possibly food-borne, microbes that move through the intestine after ingestion can be referred to by this term. Several bacterial strains, including *Lactobacillus *species, *Pediococcus acidilactici*, *Bifidobacterium bifidum*, *Bifidobacterium animalis*, and *Streptococcus thermophiles*, administered alone or in combination, have been shown to reduce CNS inflammation [115-118]. Heat-killed microorganisms have been successfully used in some situations [118,119]. Heat-killed bacteria are not considered probiotics; however, this shows that items generated from bacteria may still have therapeutic value. Since it considerably reduces the severity of EAE in mice, *Bacteroides fragilis* does indeed serve as a probiotic [109,120], but its outer membrane vesicles can also have immunomodulatory effects and prevent inflammation [109,121].

Clinical applications

Nevertheless, there is a critical need for new strategies for preventing CNS deterioration. Given the well-established relationship between gut bacteria and brain physiology, new medicines that target the gut microbiome are being developed. Therefore, new methods for treating MS and other autoimmune diseases in humans that focus on modulating the microbiota may herald a significant paradigm change in our understanding of how to treat these diseases.

Dietary Modification

Modulation of the gut microbiome can occur without external intervention or treatment administered orally by simply altering one's diet. A calorie-restricted diet has been reported to help reduce the symptoms of EAE, whereas a high-salt diet can worsen the condition by promoting Th17 differentiation [84,85,122]. In individuals with relapsing-remitting MS, prior studies have discovered a favorable association between salt consumption, exacerbation rates, and radiological activity [87,109]. Interestingly, studies in GF mice fed an elemental diet suggest that dietary Ags can affect immunity independently of gut flora [109,123].

Overall, more fiber-rich diets are linked to higher microbial diversity [109,124]. In particular, the Firmicutes and Bacteroidetes groups of bacteria are supported by a high-fiber diet. These microorganisms are involved in the fermentation process that breaks down non-digestible fibers in the colon and creates SCFAs. Short-chain fatty acids such as propionate, acetate, and butyrate are essential for reducing inflammation by stimulating Tregs [125]. A diet high in long-chain fatty acids, on the other hand, can encourage Th17 differentiation and aggravate illness [88].

Polysaccharide A Therapy

Polysaccharide A (PSA), a single capsular polysaccharide, was found to be essential for *Bacteroides fragilis* probiotic activity [120]. When given to mice via oral gavage, PSA extracted from *Bacteroides fragilis* can prevent and treat EAE [126]. In a toll-like receptor 2 (TLR2)-dependent manner, *Bacteroides fragilis* and PSA can both train DCs to produce Tregs [120,121,126-128]. This increase in Treg activity suggests that supplementation with PSA may be an appealing new treatment option for MS patients. Importantly, mouse models of depression and autism spectrum disorder, as well as autoimmunity, show that gut microorganisms and microbial metabolites can affect CNS activity, confirming the significance of the gut-brain axis [129-131].

## Conclusions

The so-called gut-brain axis has been shown to have a significant impact on host neuromodulation, endocrine functions, and modulation of host immune responses, which is crucial for controlling intestinal, systemic, and brain inflammation (neuroinflammation). Changes in the gut microbiota, i.e., dysbiosis, affect the immune system, resulting in autoimmune disorders such as multiple sclerosis and inflammatory bowel disease. In patients with AIDs, the gut epithelial barrier is frequently dysfunctional, and increased permeability promotes bacterial translocation. There is a need to ascertain the long-term safety of such therapeutic interventions because the use of probiotics is expanding tremendously. Future strategies include fecal microbiota transplantation (FMT) and targeted immunotherapies based on baseline microbiome composition.
